# Minimally important change and smallest detectable change of the OSTRC questionnaire in half‐ and full‐marathon runners

**DOI:** 10.1111/sms.13885

**Published:** 2021-03-23

**Authors:** Thierry P. C. Franke, Henrica C. W. de Vet, Bionka M. A. Huisstede

**Affiliations:** ^1^ Department of Rehabilitation, Physical Therapy Science & Sport, Brain Center, University Medical Center Utrecht Utrecht University Utrecht The Netherlands; ^2^ Department of Epidemiology and Data Science Amsterdam Public Health Research Institute Amsterdam University Medical Centers, Location VUmc Amsterdam The Netherlands

**Keywords:** interpretability, measurement error, Oslo sports trauma research center (OSTRC), running [Mesh], running‐related injuries

## Abstract

The purpose of this study was to evaluate the smallest detectable change (SDC), minimally important change (MIC), and factor structure of the Oslo Sports Trauma Research Center (OSTRC) questionnaire severity score in half‐ and full‐marathon runners. Data came from a prospective cohort study, the SUcces Measurement and Monitoring Utrecht Marathon (SUMMUM) 2017 study. Two external anchors, the global rating of change (GRC) and global rating of limitations (GRL), were used to classify the running‐related injuries (RRI) as truly improved, unchanged, or truly worsened. SDC values were calculated at individual and group levels. MIC values were calculated using the visual anchor‐based MIC distribution and mean change methods. Confirmatory factor analysis (CFA) was used to study the a priori hypothesized factor structure. A total of 132 runners who reported the same RRI on two occasions 2 weeks apart were included in the analysis. SDC values at individual and group levels were ≤35.06 and ≤9.30, respectively. With the visual anchor‐based MIC distribution method, the MIC values for RRIs that truly improved according to the GRC and GRL anchors were 13.50 and 18.50, respectively. With the mean change method, the MIC values for RRIs that truly improved according to the GRC and GRL anchors were 15.49 and 45.38, respectively. The CFA confirmed that the OSTRC was a unidimensional questionnaire. The change score of the OSTRC severity score can be used to distinguish between important change and measurement error at a group level using the MIC value 18.50. Because the SDC of the OSTRC severity score was larger than the MIC, it is not advised to use the MIC at an individual level.

**Key messages**

1. **What are the new findings**
2. The smallest detectable change, that is, the smallest change in score beyond measurement error, of the OSTRC severity score for individual and groups of marathon runners is ≤35.06 and ≤9.30, respectively.
3. The minimally important change, that is, the smallest change score which is truly important to the runner, of the OSTRC severity score is 18.50 for injured (half) marathon runners, considering both the GRC and GRL anchors.
4. The change score of the OSTRC severity score can be used at a group level to differentiate between truly important change and measurement error for, per example, research purposes.
5. The change score of the OSTRC severity score might not be suited for use at an individual for individual runners to differentiate between important change and measurement error, because the smallest detectable change is larger than the minimally important change.
6. The Dutch version of the OSTRC has a unidimensional factor structure, which supports its structural validity

## 1 INTRODUCTION

In the Netherlands, over two million people participated in running as a sport in 2014.^1^ These runners had 710 000 running‐related injuries (RRI), which resulted in €2.9 million direct medical costs, and €5.4 million in costs due to work absenteeism.

In 2014, the Athletics Consensus Group published a consensus statement on the health‐related surveillance of injuries and illness in athletes, some of whom were half‐ and full‐marathon runners.^2^ The Group advised repeated assessment of a runner's injury status over time in order to detect injuries, including RRIs, that do not cause time loss from running, but which do lead to a reduced training intensity or duration, or which cause pain during running in half‐ and full‐marathon runners. Specifically, the Group proposed using the Oslo Sports Trauma Research (OSTRC) questionnaire on health problems to register these injuries in athletics.^3^

The OSRTC is an easy‐to‐use questionnaire, consisting of four questions scored on a Likert scale (Appendix 1).^3^, ^4^ The OSTRC reflects the impact of health problems (ie, injuries or illness symptoms) on participation, training volume, sports performance, and symptoms as reported by the athlete. The sum of the four answer scores, the OSTRC severity score, is used to measure and monitor the severity of the health problem. The OSTRC was specifically designed, in cooperation with athletes, to record health problems in large heterogeneous groups of athletes.^3^

Several studies have confirmed that the OSTRC has adequate face validity to register and monitor health problems in athletes from a variety of sports.^3^, ^4^, ^5^ Moreover, the OSTRC has been translated and validated (content validity,^6^, ^7^ face validity^8^) in several languages. The Danish version of OSTRC was validated in a study population that included numerous runners.^8^ In addition, as the OSTRC measures general aspects of injury and disease, it is assumed to have face validity for half‐ and full‐marathon runners.

Thus, changes in the OSTRC severity score should reflect actual changes in the impact of the RRI. However, it can only be established whether the OSTRC severity score truly changes if its smallest detectable change (SDC) and minimally important change (MIC) are known.^9^ The SDC reflects the smallest change in OSTRC severity score that can be considered as true change, that is, not measurement error,^9^ and the MIC is the smallest change in OSTRC severity score that is truly relevant to the runner.^10^

For example, if the OSTRC is filled in twice, then the change in the OSTRC severity score could be used to monitor recovery from the RRI. In this way, changes in the OSTRC severity score inform runners, trainers, or sports clinicians, that is, at an individual level. In research, the OSRTC could be used as outcome measure in an RCT. In that case, the OSTRC is used at a group level, comparing the mean OSTRC severity score between intervention and control arms. Hence, from a clinimetric perspective, it is important to know whether the change in OSTRC severity score is greater than the MIC. Therefore, the purpose of this study was to determine the SDC and MIC of the OSTRC severity score in half‐ and full‐marathon runners with RRIs. As the unidimensionality of the OSTRC has not been assessed before, we performed a confirmatory factor analysis (CFA) to determine the structural validity, as part of construct validity, of the OSTRC questionnaire.

## 2 METHODS

### 2.1 Design

Data from a prospective cohort study, the SUcces Measurement and Monitoring Utrecht Marathon (SUMMUM) 2017 study, were used. The study was approved by the University Medical Center Utrecht ethics committee (protocol number 16/503).

### 2.2 Participants

All runners who registered to participate in the half or full Utrecht Marathon (UM) from September 1st, 2016, up to March 19th, 2017, (date of the UM) were asked if they were interested in participating in the study. Runners were recruited during registration for the UM via a newsletter or during a symposium on RRIs. Interested runners were sent an information letter. They provided informed consent before filling in the baseline questionnaire. Runners were included if they (a) were 18 years or older; (b) had an e‐mail address; and (c) had adequate Dutch language skills.

### 2.3 Procedures

Every 4 weeks during the registration period, a new group of runners entered the study. Data collection for the first group started on November 25th, 2016, 16 weeks before the UM. Group 2 entered the study on December 22nd, 2016, (12 weeks before the UM), group 3 on January 20th, 2017, (8 weeks before the UM), group 4 on February 17th, 2017, (4 weeks before the UM), and group 5 on March 20th, 2017 (the day after the UM).

All groups of runners started by filling in the baseline questionnaire. Subsequently, every 2 weeks questionnaires were sent to the runners up to the date of the UM. The day after the UM, the runners completed the post‐marathon questionnaire regarding their participation in the UM. Group 5 only received the baseline and post‐marathon questionnaires. Runners had 7 days to complete the baseline questionnaire and 5 days for all subsequent questionnaires. Reminders were sent if runners failed to complete a questionnaire. All questionnaires were made in NetQ (NetQuestionnaires, NetQ Healthcare BV, Amsterdam, The Netherlands) and were sent via e‐mails containing a hyperlink to the web‐based questionnaires.

### 2.4 Questionnaires

#### 2.4.1 Baseline questionnaire

Demographic data on the runners were taken from the baseline questionnaire.

#### 2.4.2 OSTRC

We used the Dutch version of the OSTRC on health problems.^11^ The OSTRC was translated into Dutch using a forward‐backward translation as described by Beaton et al.^12^ While the OSTRC can be used to monitor the impact of both RRIs and illness symptoms, in this study we used it to establish whether runners had an RRI and to monitor the impact of the RRI (Appendix 1). As described by Clarsen et al, the OSTRC consists of four questions, of which the summed answer scores are used to calculate the OSTRC severity score (range 0‐100, a higher score indicates a higher severity).^3^

An exploratory factor analysis of the Dutch version of the four OSTRC questions showed one underlying latent construct for the four OSTRC questions and adequate internal consistency (Cronbach's alpha 0.91).^11^ If the severity score was >0, a runner was considered to be injured and was asked follow‐up questions about the anatomical location, type, and duration of the RRI. A RRI was any self‐reported complaint involving muscles, joints, tendons, and/or bones considered by the runner to be caused by running.^11^ If the same RRI was registered on two consecutive occasions (based on location, type, and duration of the RRI), the OSTRC severity change score was calculated by subtracting the score of the second OSTRC measurement from the first one.

#### 2.4.3 Anchor questionnaires

To evaluate the MIC and SDC of the OSTRC severity score, external criteria were used to determine whether runners’ RRI status changed over time. Because the MIC depends on the methodology used, two anchor questions were used^13^: the global rating of change (GRC) and the global rating of limitations (GRL). If no RRI was reported, runners were asked if they had reported an RRI 2‐4 weeks ago. If so, runners were asked to complete the GRL and GRC.

The GRC, a retrospective anchor, was used to study the change in impact of the RRI during the last two weeks compared to when the runner first perceived this RRI. The GRC anchor inherently contains the change in the impact of the RRI. The runner could fill in one of the following seven answers: “very much worse,” “much worse,” “slightly worse,” “unchanged,” “slightly improved,” “much improved,” and “very much improved.” RRIs were classified as truly improved if runners answered “much improved” or “very much improved.” RRIs were considered to have become truly worse if runners answered “much worse” or “very much worse.” RRIs were considered to be unchanged if runners answered “slightly worse,” “unchanged,” or “slightly improved.” This was done to avoid socially desirable answers and to ensure that the measured change was clinically important.

The GRL was used as a five‐point prospective anchor and asks runners to rate their limitations in running performance due to the reported RRI. Use of a prospective anchor decreases the risk of recall bias. Possible answers were “poor,” “fair,” “moderate,” “good,” and “excellent” (scored 1, 2, 3, 4, and 5, respectively). A change score was calculated by subtracting the second GRL score from the first one. A runner's RRI was considered to have truly improved or worsened if the GRL score changed ≥2 points.

### 2.5 Statistical analysis

The baseline characteristics of the half‐ and full‐marathon runners were described using descriptive statistics and were compared using a chi‐squared test (categorical variables) and *t* test (continuous variables). In the case of a non‐normal distribution, the Mann‐Whitney *U* test was used.

Runners’ data were included in the SDC and MIC calculations if (a) the same RRI was registered with the OSTRC on two consecutive occasions (if the anatomical locations matched and the RRI duration was 2 weeks or longer, or if the OSTRC severity score was zero and a RRI was reported 2 weeks ago, on the previous OSTRC); and (b) both the GRL and GRC anchor questionnaires had been completed.

Regarding the desired sample size, the recommendations of the COSMIN checklist were followed (n = 100) because no clear guidelines exist on sample size calculations for studies determining MIC values.^14^ A priori the significance level was set at *P* =.05. The SDC and MIC analyses were performed using SPSS (v.21, IBM, Armonk, New York, USA.)

### 2.6 Smallest detectable change

The SDC is the smallest change in OSTRC severity score that can be considered a true change, that is, change beyond the measurement error.^9^ Knowledge of the SDC of the OSTRC severity score provides a data‐driven estimate of whether there is a change over and above chance. SDC calculations require a stable sample, that is, no change in RRI impact. Therefore, the SDC was calculated for runners with a GRL change score of zero and for runners with a GRC score of “unchanged.” A two‐way mixed ICC_agreement_ was used to calculate the mean square observer and mean square error.^10^ Subsequently, the standard error of the measurement (SEM) was calculated as the square root of the sum of the mean square observer and mean square error. The SDC was calculated for individual [SDC_individual_ = 1.96 × √2 × SEM] and groups [SDC_group_ = SDC_individual_/√n] of athletes.

### 2.7 Minimally important change

The MIC is the smallest change in OSTRC severity score that is truly relevant to the runner.^10^ Because MIC values can vary by how they are calculated, two anchor‐based methods were used: the visual anchor‐based MIC distribution method and the mean change method.^10^, ^15^ The MIC was calculated for RRIs that truly improved. To monitor changes in self‐reported assessment of the impact of the RRI, the SDC of the OSTRC severity score should be smaller than the MIC.^16^

#### 2.7.1 Visual anchor‐based MIC distribution method

Receiver operator characteristic (ROC) curves were plotted to calculate the optimal MIC and the area under the curve (AUC; 95% confidence interval [CI]) for RRIs that truly improved (the GRC categories “much improved,” or “very much improved”) according to the anchor questionnaires. The optimal MIC was the point on the ROC curve where the sum of [1‐sensitivity] and [1‐specificity] was the smallest, yielding the smallest amount of misclassification.^10^ To reflect the uncertainty of the MIC estimation, a 95%CI upper limit was calculated [mean change + 1.645 × SD_change_], based on the runners whose RRIs were unchanged according to the anchors.^16^ The AUC reflects the ability of the OSTRC severity score to correctly identify injured runners whose RRI has truly changed.^10^ An AUC value > 0.70, with a 95% CI lower limit > 0.50, is considered to be discriminatory.^17^

Furthermore, to visualize the distribution of the OSTRC severity change scores, two‐sided graphs were plotted for runners whose RRIs had truly improved, showing the OSTRC severity change score and the proportional frequency (number of runners with a specific OSTRC severity change score divided by the total number of runners) on the *y*‐axis and *x*‐axis, respectively. The proportional frequency of runners whose RRIs had truly improved and runners whose RRIs were unchanged were plotted on the left and right sides of the *y*‐axis, respectively.

#### 2.7.2 Mean change method

On the basis of data for runners whose RRIs truly improved (ie, GRC score = “much improved” and GRL change score = 2), two MIC values were calculated as the mean change scores (95% CI) of the OSTRC severity score.

### 2.8 Anchor suitability

Spearman's correlation was calculated for the OSTRC severity change score and the GRL and GRC anchors to determine whether the latter questionnaires measured the same change in the impact of the RRI as was measured with the OSTRC severity score (i.e., *r* ≥ .50).^18^

### 2.9 Factor structure to support structural validity

In order to assess the a priori hypothesized factor structure of the OSTRC questionnaire, a CFA was performed. The authors hypothesized that the OSTRC is a unidimensional questionnaire. This hyposthesis was based on previous studies reporting a single underlying latent variable from the OSTRC using exploratory factor analysis.^4^, ^11^ Thus, a 1‐factor model was fitted. The CFA was performed using a diagonally weighted least squares analysis because the OSTRC question answers are categorical. The following parameters and criteria were used to indicate an adequate model fit: chi‐square with *P*‐value >.05, robust comparative fit index (CFI) > 0.95, root‐mean‐square error of approximation (RMSEA) < 0.06, and a standardized root‐mean‐square residual (SRMR) of <0.08.^19^ The CFA was performed in R (version 1.2), using the package Lavaan.^20^

## 3 RESULTS

### 3.1 Sample characteristics

Of 1084 runners invited to participate in this study, 538 met the inclusion criteria (Appendix 2). Of these runners, 132 reported the same type of RRI on at least two consecutive OSTRCs and completed the GRL and GRC questionnaires. Their data were included in the statistical analysis. Of these 132 runners, 105 and 27 participated in the half and full UM, respectively. No significant differences were found between the half‐ and full‐marathon runners in terms of sex (*P* =.119), age (*P* >.070), height (*P* =.447), or weight (*P* >.769; Table 1). Knee (27%) and lower leg (14%) RRIs were the most common RRIs and were mainly overload (35%) and “muscle or tendon” (32%) RRIs. In most cases, the duration of the RRI was 2‐4 weeks (33%), or longer than 8 weeks (25%).

**TABLE 1:** Baseline characteristics

	All runners (n = 132)	Half marathon (n = 105)	Full marathon (n = 27)	*P*‐value*
Sex, F/M, n (%)	60/72 (45/55)	51/54 (49/51)	9/18 (33/67)	.156
Age, y, (mean ± SD)	38.3 ± 11.0	37.7 ± 11.3	40.9 ± 9.5	.105
Height, cm, (mean ± SD)	177.0 ± 9.5	176.8 ± 9.5	178.1 ± 9.5	.395
Weight, kg, (mean ± SD)	70.8 ± 11.2	71.0 ± 11.7	70.0 ± 9.4	.959
Anatomical location of the RRI, n (%)				
Cervical spine	1 (1)	1 (1)	0 (0)	
Chest, ribs	1 (1)	1 (1)	0 (0)	
Thoracic spine	2 (2)	2 (2)	0 (0)	
Lumbar spine	12 (9)	11 (11)	1 (4)	
Pelvic floor	1 (1)	0 (0)	1 (4)	
Upper extremity	1 (1)	1 (1)	0 (0)	
Hip	7 (5)	6 (6)	1 (4)	
Groin	3 (2)	2 (2)	1 (4)	
Gluteal region	3 (2)	2 (2)	1 (4)	
Upper leg	4 (3)	4 (4)	0 (0)	
Dorsal side upper leg or hamstring	9 (7)	6 (6)	3 (11)	
Knee	36 (27)	31 (30)	5 (19)	
Lower leg	17 (13)	12 (11)	5 (19)	
Achilles tendon	9 (7)	7 (7)	2 (7)	
Ankle	11 (8)	7 (7)	4 (15)	
Foot/toe	15 (11)	12 (11)	3 (11)	
Type of RRI, n (%)				
Contusion	3 (2)	2 (2)	1 (4)	
Distortion	9 (7)	7 (7)	2 (7)	
Muscle or tendon	41 (31)	33 (31)	8 (30)	
Fracture	2 (2)	2 (2)	0 (0)	
Chondral injury	2 (2)	2 (2)	0 (0)	
Back injury	9 (7)	9 (9)	0 (0)	
Hernia	3 (2)	2 (2)	1 (4)	
Bursitis	2 (2)	2 (2)	0 (0)	
Overload	46 (35)	35 (33)	11 (41)	
Other	10 (8)	6 (6)	4 (15)	
Duration of the RRI, wk, n (%)				
0‐2 wk	30 (22)	22 (21)	8 (30)	
2‐4 wk	44 (33)	37 (35)	8 (26)	
4‐6 wk	17 (13)	12 (11)	4 (14)	
6‐8 wk	9 (7)	8 (7)	1 (4)	
More than 8 wk	32 (24)	26 (25)	7 (26)	

Note

Percentages are rounded to the nearest whole number; therefore, the sum might not be 100%.

Abbreviations: ∆, change score; cm, centimetre; F, female; kg, kilogram; M, male; RRI, running‐related injury.

* Tests performed between half‐ and full‐marathon runners.

### 3.2 OSTRC and anchor questionnaire responses

Table 2 shows the OSTRC severity change scores for the GRC and GRL anchor questionnaires per answer category. The number of runners whose injury truly worsened according to the anchors was small. Therefore, we did not calculate MIC values for runners whose RRI truly worsened.

**TABLE 2:** OSTRC severity change scores per anchor answer category

	All runners n (%)	OSTRC severity score Mean difference	SD
GRC anchor score			
Very much worse	0 (0)	‐	‐
Much worse	0 (0)	‐	‐
Slightly worse	13 (10)	−2.08	32.17
Unchanged	14 (11)	5.14	19.47
Slightly improved	44 (33)	0.32	21.26
Much improved	37 (28)	15.49	24.02
Very much improved	24 (18)	30.17	24.92
GRL ∆			
−3	2 (2)	−69.00	4.24
−2	5 (4)	−11.20	35.19
−1	11 (8)	−15.36	26.55
0	62 (47)	7.35	16.87
1	39 (30)	18.49	19.17
2	7 (5)	44.00	22.77
3	6 (5)	39.00	29.75

Abbreviations: ∆, change score; GRC, Global Rating of Change Anchor; GRL, Global rating of Limitations Anchor; OSTRC, Oslo Sports Trauma Research Center Questionnaire; SD, standard deviation.

### 3.3 Smallest detectable change

Table 3 shows the SDC of the OSTRC severity score at individual and group levels for runners with a GRL change score of zero and for runners with a GRC score “unchanged.”

**TABLE 3:** Smallest detectable change

	Runners with ∆ GRL = 0 (n = 62)	Runners with GRC = unchanged (n = 14)
∆ OSTRC severity score (95% CI)	−7.68 (−11.86; −3.50)	−5.14 (−15.34; 5.06)
SEM_agreement_ ^a^	11.97	12.55
SDC at an individual level^b^	35.06	34.78
SDC at a group level^c^	4.45	9.30

Abbreviations: ∆, change score; 95% CI, 95% confidence interval; GRC, Global Rating of Change Anchor; GRL, Global rating of Limitations Anchor; OSTRC, Oslo Sports Trauma Research Center; SDC, smallest detectable change; SEM, standard error of the mean.

^a^ SEM_agreement_ = √(mean square observer + mean square error).

^b^ SDC_individual_ = 1.96 × √2 × SEM_agreement_

^c^ SDC_group_ = SDC_individual_/√n

### 3.4 Minimally important change

#### 3.4.1 Visual anchor‐based MIC distribution method

**FIGURE 1:**
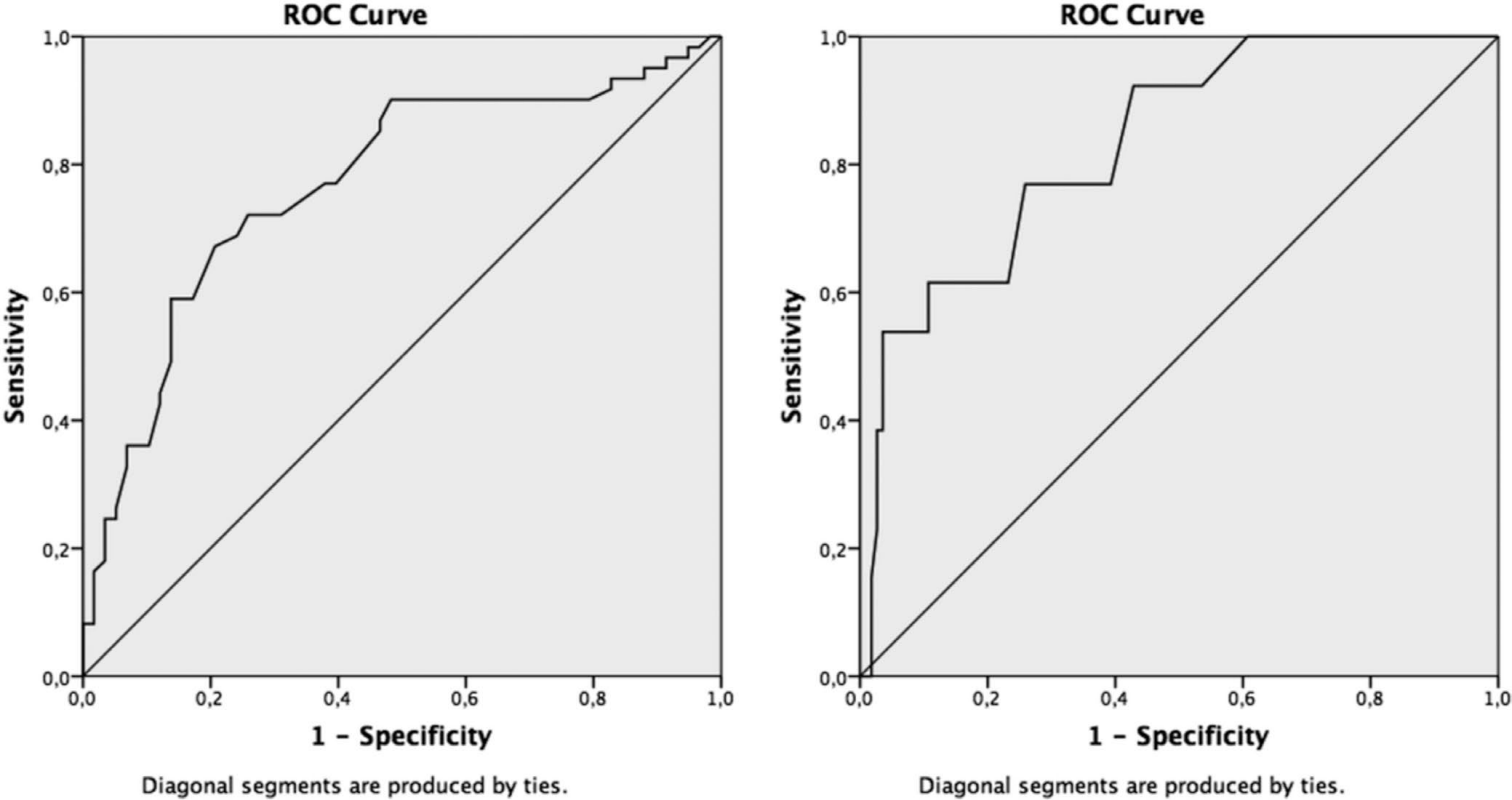
Receiver operator characteristic (ROC) curves. **Left** ROC curve for runners with running‐related injuries whose injury had improved according to the global rating of change (GRC) anchor; **right** ROC curve for runners with running‐related injuries whose injury had improved according to the global rating of limitations (GRL) anchor

**FIGURE 2:**
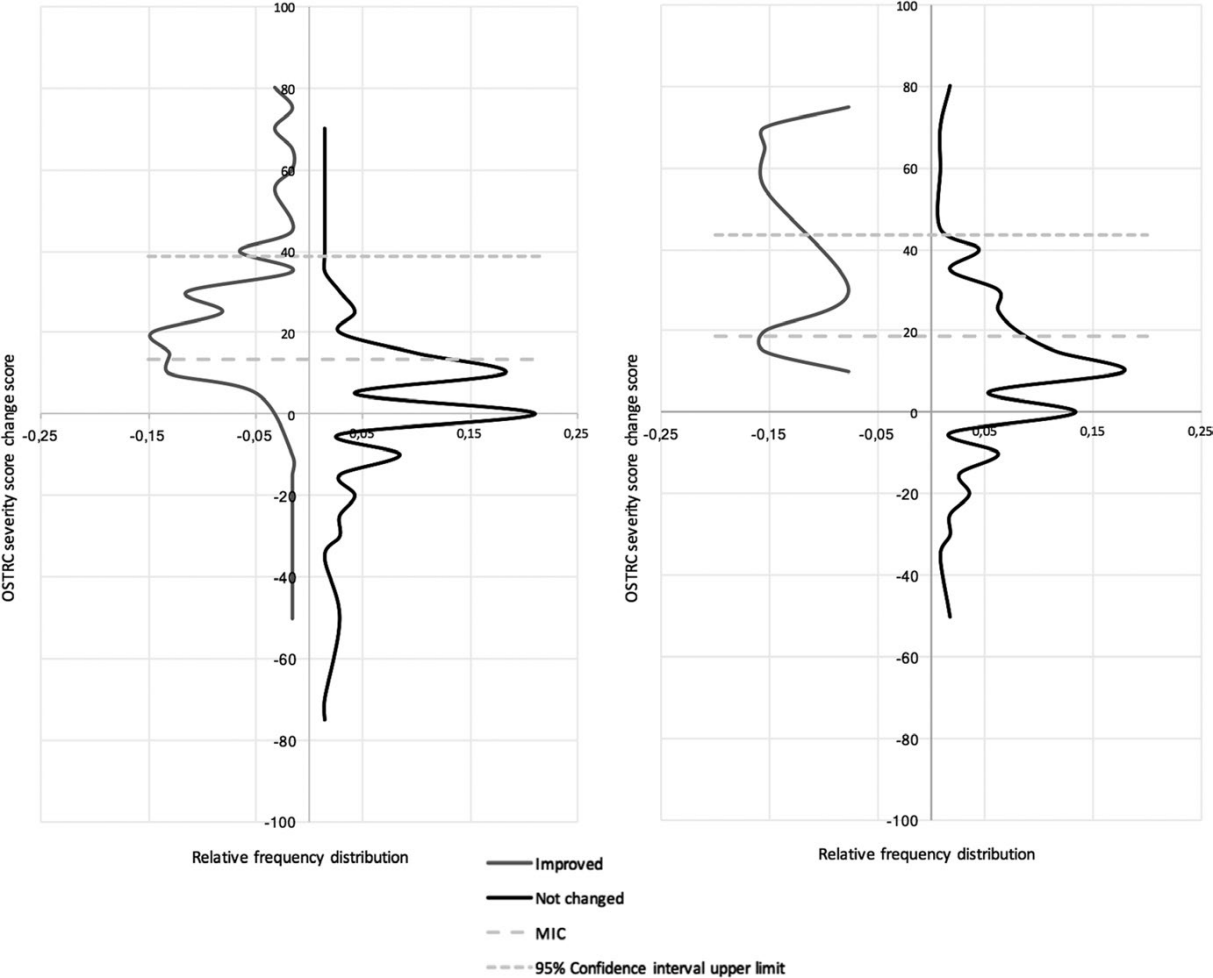
Visual anchor‐based MIC distribution according to the global rating of change (GRC) anchor (left) and global rating of limitations (GRL) anchor (right). **Left** **graph** MIC according to the GRC anchor (MIC cut‐off = 13.50 points, 95% confidence interval upper limit 38.74); grey line, distribution of OSTRC scores of runners whose RRI had improved according to the GRC anchor; black line, distribution of OSTRC scores of runners whose RRI was unchanged according to the GRC anchor; grey dotted line, MIC cutoff value and the 95% confidence interval upper limit. **Right graph** MIC according to the GRL anchor (MIC cutoff 18.50 points, 95% confidence interval upper limit 43.56); Grey line, distribution of OSTRC scores of runners whose RRI had improved according to the GRL anchor; black line, distribution of OSTRC scores of runners whose RRIs were unchanged according to the GRL anchor; Grey dotted line, MIC cutoff value and the 95% confidence interval upper limit

For runners whose RRI truly improved, as assessed with the GRC anchor (n = 62), the AUC (95% CI) of the ROC curve and the optimal MIC for the OSTRC severity score (95% CI upper limit) were 0.77 (0.69‐0.86; Figure 1) and 13.50 (38.74; Table 4 and Figure 2), respectively. For runners whose RRIs had truly improved, as assessed with the GRL anchor (n = 13), the AUC and the optimal MIC were 0.83 (0.77‐0.94) and 18.50 (43.56), respectively.

**TABLE 4:** Minimally important change

			MIC ± SD	95% CI	AUC (95% CI)	Sensitivity (%)^a^	Specificity (%)^b^
Visual anchor‐based MIC distribution	GRC anchor	Truly improved (n = 61)	13.50^c^	38.74^d^	0.77 (0.68‐0.86)	67.2	79.3
	GRL Anchor	Truly improved (n = 13)	18.50^c^	43.56^d^	0.83 (0.73‐0.94)	76.9	74.1
Mean change method	GRC Anchor	Truly improved (n = 37)	15.49 ± 24.02	(7.48‐23.49)			
	GRL Anchor	Truly improved (n = 7)	44.00 ± 22.78	(22.94‐65.06)			

Abbreviations: ‐, item is not applicable; 95% CI, 95% confidence interval; AUC, area under the curve; GRC, Global Rating of Change; GRL, Global Rating of Limitations; MIC, minimally important change; OSTRC, Oslo Sports Trauma Research Center; SD, standard deviation.

^a^ sensitivity is the percentage of runners whose RRI is correctly classified as improved, using the MIC value chosen.

^b^ specificity is the percentage of runners whose RRI is correctly classified as unchanged, using the MIC value chosen.

^d^ only upper limit of the 95% CI was selected as proposed by de Vet et al (2007)[14].

^c^ Using the visual anchor‐based MIC distribution method, no SD could be calculated.

#### 3.4.2 Mean change method

Runners whose RRIs had truly improved, as assessed with the GRC (n = 37) and the GRL (n = 7) anchors, had a mean change in OSTRC severity score of 15.49 (95% CI 7.48‐23.49) and 44.00 (95% CI 22.94‐65.06), respectively (Table 4).

### 3.5 Anchor suitability

The Spearman's correlation coefficient of the OSTRC severity score and the GRL change score (*r* = .53) exceeded the predetermined criterion (*r* ≥ .50). However, the correlation between the GRC score (*r* = .49) did not exceed the predetermined criterion. Thus, the GRL anchor was suitable to establish the change in the impact of the RRI, whereas the GRC anchor might not have been suitable.

### 3.6 Factor structure to support structural validity

A CFA was conducted to evaluate whether a 1‐factor model fits the OSTRC questionnaire. The fit parameters were chi‐square = 1.77, Degrees of freedom = 2, *P*‐value = .41, CFI = 1.00, RMSEA = 0.00 (90% CI 0.00; 0.17), and SRMR 0.02. The factor loadings of the individual OSTRC questions on the latent variable were question (a) 1.00, question (b) 1.06, question (c) 0.95, and question (d) 0.60. So, the CFA indicated an adequate model fit and therefore adequate structural validity of the OSTRC. Moreover, the hypothesized unidimensionality was confirmed.

## 4 DISCUSSION

This study evaluated the MIC and SDC of the OSTRC severity score for RRIs in half‐ and full‐marathon runners. We concluded that if an RRI is registered twice over a two‐week period, the OSTRC severity score can be used to distinguish important change from measurement error at a group level because the SDC is smaller than the MIC. Such analyses are often performed in research settings. However, at an individual level, the SDC is larger than the MIC. Therefore, the OSTRC severity change score cannot distinguish between important change and measurement error in individual half‐ or full‐marathon runners. We advise using a MIC of 18.50 to determine whether the impact of the RRI has decreased at a group level.

Further, we used CFA to evaluate the structural validity of the OSTRC questionnaire and concluded that the OSTRC is a unidimensional questionnaire. The high factor loadings and CFI and low RMSAE reported might be explained by the similar nature of the four OSTRC questions and the limited number of questions in the OSTRC. To the authors’ knowledge, no other studies have used CFA to assess the dimensionality of the OSTRC. Principal component analysis of the OSTRC showed one underlying factor,^11^ which is in line with our findings.

A plethora of methods is available for calculating MIC values. These can be roughly divided into anchor‐based and distribution‐based methods. We used two methods, one of which is the visual anchor‐based MIC distribution method, which combines the advantages of both the anchor‐ and distribution‐based methods.^16^ The second method we used, the mean change method, is an anchor‐based method. Anchor‐based methods have a higher validity than distribution‐based methods, because the anchors inherently assess the importance of the change.^10^, ^15^, ^21^ The visual anchor‐based MIC distribution method is preferred to the mean change method. As it regards the OSTRC as a diagnostic test.^10^ Using this method, we calculated an MIC of 13.50 with the GRC anchor and 18.50 with the GRL anchor. The MIC of 18.50 had a greater AUC = 0.83 (95% CI 0.73‐0.94), greater sensitivity 76.9% (Table 4), and adequate anchor suitability (GRL anchor correlation 0.53) and is thus preferred. Moreover, it is a conservative estimate because it is higher than the MIC values based on the GRC anchor. Further, we used two anchors, a retrospective anchor and a prospective anchor, because MIC calculations can vary depending on the anchors used.^13^ These two anchors are often used in research into musculoskeletal disorders and in sports medicine. Moreover, these anchors are recommended in the literature on clinimetric research.^13^, ^15^

Both versions of the OSTRC questionnaire, the OSTRC on overuse injuries and the OSTRC on health problems, use the severity score, that is, the sum of the individual answer scores.^3^, ^4^ We calculated the SDC and MIC of the OSTRC on health problems for RRIs reported by half‐ and full‐marathon runners during a preparatory period before a running event. The severity score of both OSTRC questionnaires intend to objectively measure and monitor the health status of individual and groups of athletes over time.^3^, ^4^ Thus, the OSTRC measures the progression of each individual RRI or health problem reported. However, the MIC and SDC of these questionnaires have not yet been reported in scientific literature, even though the questionnaires have been translated into several languages.^6^, ^7^, ^8^, ^11^, ^22^ Hence, it is not possible to compare our findings with those of others. Our findings caution against the use of OSTRC severity change scores in determining whether an individual runner's RRI status has truly changed. Moreover, it is not advised to use MIC values based on a single study, as the MIC might depend on the characteristics of the population and the method used to calculate it.^15^ Thus, multiple studies investigating the SDC and MIC of the OSTRC severity score are needed both in half‐ and full‐marathon runners and other athletic populations. Thereafter, systematic reviews and expert panel studies are needed to achieve consensus on the MIC in OSTRC severity score.^23^

MIC values may depend on the initial severity of a RRI.^21^ If the MIC is dependent on the initial OSTRC severity score, then perhaps the MIC should be expressed as a percentage of the initial score.^15^ However, we could not investigate this because of the limited sample size of our study.

The OSTRC on health problems was designed using classical test theory. This provides for a straightforward interpretation of the sum score, but does not enable differentiation between the sample characteristics and the characteristics of the OSTRC.^10^ Item response theory (IRT) could make this differentiation possible by more closely investigating the relationship between the questions and the latent variable the OSTRC intends to measure in order to predict the probability of certain answer scores. Future research could look into the psychometric properties of the OSTRC severity score by using IRT.

### 4.1 Limitations

This study had several limitations. A possible limitation of this study is that we included all RRI locations and types when calculating the MIC. Because the MIC might vary by the type or location of an RRI, the questions of the OSTRC might show differential item function (DIF). However, the sample size in our study was not sufficient to stratify the runners by RRI location or type in order to estimate multiple MICs or perform DIF analysis.^24^

Further, separate MIC values can be calculated for RRIs that have truly improved or worsened.^10^ In our study, there was an insufficient number of RRIs that truly worsened, so no MIC values could be estimated for these RRIs. Nonetheless, this is the first study to investigate the MIC and SDC of the OSTRC severity score.

## 5 PERSPECTIVE

This study shows that the OSTRC severity score can be used to detect improvement in the impact of RRIs at a group level in half‐ and full‐marathon runners, having adequate responsiveness, interpretability, and factor structure. We advise using a MIC of 18.50 for groups of half‐ and full‐marathon runners because of the greater AUC and sensitivity of this value compared with other MIC values. Thus, at a group level it can be concluded that the impact of an RRI has decreased if the OSTRC severity score decreases by more than 18.50 points. However, this MIC of the OSTRC severity score may not be appropriate for individual runners because the SDC was greater than the MIC at an individual level. The SDC and MIC values of the OSTRC may vary per athletic population as they are dependent on the characteristics of the athletes. Therefore, caution is warranted if our results are to be applied to other types of athletes. Future studies should determine the SDC and MIC of the OSTRC severity score for different types of athletes.

## CONFLICT OF INTEREST

The authors have no conflicts of interest to declare.

## Figures and Tables

**Figure:**
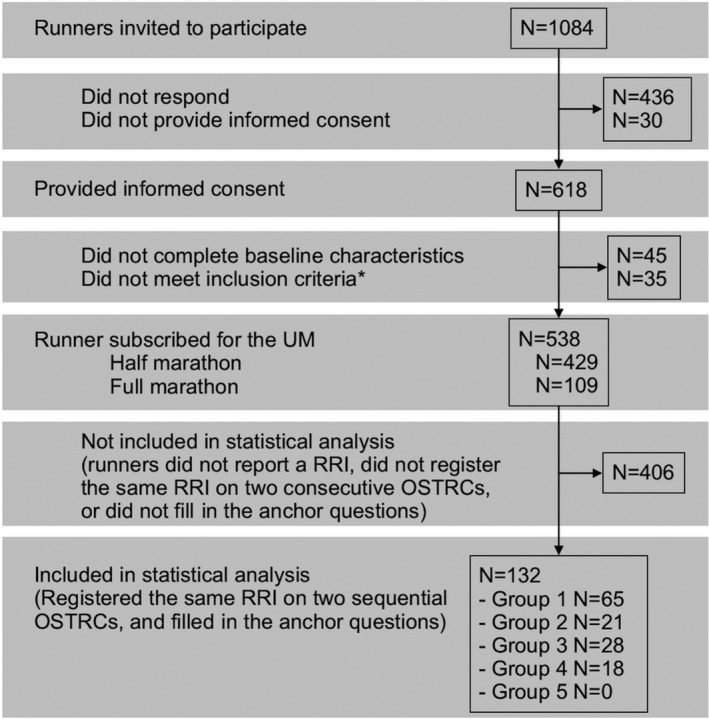
Flow of the participants during the study. Abbreviations: OSTRC, Oslo Sports Trauma Research Center Questionnaire; RRI, running‐related injury; UM, Utrecht Marathon; *runners were excluded because they registered for a distance other than the half or full marathon or participated in a half or full marathon at another event.

## APPENDIX 1

### DUTCH VERSION OF THE OSTRC QUESTIONNAIRE

#### Nederlandse versie van de Oslo Sports Trauma Research Center (OSTRC) Vragenlijst monitoren van blessures en ziektes

De volgende vragen gaan over de afgelopen twee weken. Gelieve alle vragen te beantwoorden, ongeacht of u gezondheidsproblemen heeft meegemaakt gedurende deze periode. Kies het antwoord dat het beste bij u past. In geval van twijfel, wilt u dan de meest geschikte optie selecteren.

Indien u verscheidene ziekten en/of blessures ondervindt, refereer a.u.b. naar de klacht die u het hefstigste ondervond gedurende de afgelopen 2 weken. Aan het eind van de vragenlijst krijgt u de mogelijkheid om additionele gezondheidsproblemen te rapporteren.

*Vraag 1:* Heeft u problemen ondervonden bij het uitvoeren van een training en/of wedstrijd ten gevolge van een blessure, ziekte of andere gezondheidsproblemen gedurende de afgelopen 2 weken?

**Table:**

□ Volledige uitvoering zonder gezondheidsproblemen	0
□ Volledige uitvoering, inclusief blessure/ziekte	8
□ Verminderde uitvoering vanwege blessure/ziekte	17
□ Geen uitvoering vanwege blessure/ziekte	25

*Vraag 2:* In hoeverre heeft u uw trainingsomvang moeten aanpassen ten gevolge van een blessure, ziekte of andere gezondheidsproblemen gedurende de afgelopen 2 weken?

**Table:**

□ Geen vermindering	0
□ In minimale hoeveelheid	6
□ In matige hoeveelheid	13
□ In grote hoeveelheid	19
□ Niet in staat tot uitvoering	25

*Vraag 3:* In hoeverre heeft een blessure, ziekte of andere gezondheidsproblemen effect gehad op uw prestaties gedurende de afgelopen 2 weken?

**Table:**

□ Geen effect	0
□ In minimale hoeveelheid	6
□ In matige hoeveelheid	13
□ In grote hoeveelheid	19
□ Niet in staat tot uitvoering	25

*Vraag 4:* In hoeverre heeft u symptomen/gezondheidsklachten ervaren gedurende de afgelopen 2 weken?

**Table:**

□ Geen symptomen/gezondheidsklachten	0
□ Milde symptomen/gezondheidsklachten	8
□ Matige symptomen/gezondheidsklachten	17
□ Ernstige symptomen/gezondheidsklachten	25

De OSTRC somscore berekent u door de antwoordscores per vraag bij elkaar op te tellen. Indien bij vraag 2 en/of 3 de score ≥ 13 is spreekt men van een “substantiële” gezondheidsklacht.

#### Legend

Questionnaire was reproduced with permission from Journal of Orthopaedic & Sports Physical Therapy (JOSPT) from Franke TPC, Backx FJG, Huisstede BMA, Running Themselves Into the Ground? Incidence, Prevalence, and Impact of Injury and Illness in Runners Preparing for a Half or Full Marathon. J Orthop Sports Phys Ther. 2019 Jul;49(7):518‐528.
